# Correlates of Healthy Eating in Urban Food Desert Communities

**DOI:** 10.3390/ijerph17176305

**Published:** 2020-08-29

**Authors:** Allison Karpyn, Candace R. Young, Zachary Collier, Karen Glanz

**Affiliations:** 1 Acting Director, Center for Research in Education and Social Policy (CRESP), Human Development and Family Science, University of Delaware, Newark, DE 19716, USA; 2 Founder and Principal, New Leaf Consulting, LLC, Allentown, PA 18104, USA; candace@newleafconsulting.org; 3 Educational Statistics and Research Methods, School of Education and Center for Research in Education and Social Policy, University of Delaware Newark, Newark, DE 19716, USA; collierz@udel.edu; 4 George A. Weiss University Professor, Perelman School of Medicine and School of Nursing, University of Pennsylvania, Philadelphia, PA 19104, USA; kglanz@upenn.edu

**Keywords:** food access, food deserts, low-income populations, nutrition environment, community nutrition, healthy eating index

## Abstract

The food environment is well documented as an important emphasis for public health intervention. While theoretical models of the relationship between the food environment and dietary outcomes have been proposed, empirical testing of conceptual models has been limited. The purpose of this study was to explore which factors in nutrition environments are significantly associated with dietary outcomes in two urban, low-income, and minority food desert communities. This study analyzed cross-sectional data based on 796 participants from the Food in Our Neighborhood Study. Participants were recruited based on a random sample of addresses in neighborhood study areas, Philadelphia, PA (*n* = 393) and Trenton, NJ (*n* = 403). Main dietary outcomes were Healthy Eating Index (HEI) scores and fruit and vegetable consumption subscores computed from ASA24^®^ assessments. Exploratory factor analysis was conducted and yielded a model of four factors with 22 items. Among four factors that emerged, three factors (Perceptions of Neighborhood Food Availability; and Household Food Challenges) were significantly correlated with dietary outcomes. My Store’s Quality and Perceptions of Neighborhood Food Availability were positively correlated with vegetable consumption subscore. The Household Food Challenges factor was negatively correlated with both vegetable subscore and overall HEI score (i.e., more household challenges were associated with lower dietary scores). These findings confirmed the importance of perceived nutrition environments and household food challenges in predicting dietary outcomes among residents of two urban, low-income, and minority food desert communities.

## 1. Introduction

In an effort to respond to the persistent and costly prevalence of diet-related disease, researchers and practitioners have embraced models of public health intervention that include shifting focus to understand how environments where we live, work, and play impact behaviors and health. One such area is the food environment [1].

Food deserts have received considerable attention, with the United States Department of Agriculture (USDA) estimating that 19 million Americans (6.2% of the U.S. population in 2015) live in low-income and low food access census tracts [2]. Areas where socioeconomic challenges coincide with a lack of food access have stimulated public health policy, systems, and environmental strategies that specifically seek to expand healthy food access [3], and/or promote healthier in-store environments [4]. Such interventions include: healthy food financing initiatives to stimulate development of supermarkets in underserved communities; efforts to improve corner store product mix; pricing and marketing strategies; policy changes to minimum stocking requirements for Supplemental Nutrition Assistance Program (SNAP)-authorized retailers; and nutrition education in schools and retail environments [5].

Conceptual models guide research on factors that explain how nutrition environments contribute to eating behaviors. In the past decade, these models have applied an ecological framework to diet-related behavior and shifted from a purely individual focus to greater recognition that individuals interact with a complex set of social, cultural, economic, physical, and macro-level environments [6,7]. Despite the usefulness of such models, few have been tested empirically at multiple levels. Thus, the relative contribution of different aspects of the community and consumer nutrition environments on shopping behaviors, home food availability, and dietary patterns are not well articulated due to this lack of empirical research.

More recent models proposed by Green and Glanz (2015) created frameworks for a deeper understanding of the interplay between elements of community, consumer, and home food environments [8]. However, to date, empirical testing of these models has been limited, and as such, the inter- and intra-dependent relationships between neighborhood characteristics, psychosocial factors, perceptions of community food environments, home shopping behaviors, food access, and dietary outcomes are still emerging. This research seeks to address these gaps by assessing the relative contributions of critical factors of the food environment on healthy eating among residents of urban food deserts. The study design uses a multi-level application of the conceptual approach, with both person/household-level and community/store-level data. Specifically, the goal of the present analysis was to examine relative contributions of multi-level factors in the nutrition environment (i.e., perceived and observed; community, store, and household levels) on dietary outcomes in two urban, low-income, and minority food desert communities in the Northeastern United States.

This research advances the understanding of pathways in underserved communities by both testing conceptual models of perceived and observed nutrition environments in a food desert context, and examining the extent to which multi-level factors articulated in the framework contribute to fruit and vegetable intake and healthy eating measures among residents in urban food desert communities. Improved understanding of these dietary pathways is important to support frameworks for intervention in order to ultimately reduce health disparities.

## 2. Materials and Methods

Data for these analyses are from baseline surveys and contextual data from the Food in Our Neighborhood Study (FIONS). FIONS is a 5-year quasi-experimental natural experiment conducted in two urban, low-income, and minority communities—one in Philadelphia, PA and one in Trenton, NJ—both designated as “low access to supermarket” areas by USDA’s 2015 Food Environment Atlas [9]. Neighborhood study areas were matched on socio-demographic characteristics and each comprised a contiguous three-square mile area. Cross-sectional baseline data were collected in 2017, prior to the construction and opening of a new full-service supermarket in the Philadelphia study community in early 2018 [10]. The study protocol was approved by the Institutional Review Board of University of Delaware.

### 2.1. Sample and Data Collection Procedures

A random sample of 2439 addresses (*n* = 1264 in Philadelphia; *n* = 1175 in Trenton) was selected from a Computerized Delivery Sequence File database of residential addresses serviced by the U.S. Postal Service and purchased from Marketing Systems Group, an address based vendor [11]. A sample of approximately 1200 randomly sampled addresses was established in each study area in order to ensure a final sample of at least 600 participants after attrition, a figure that would power the study to compare and detect changes in dietary outcomes over time.

**Figure 1:**
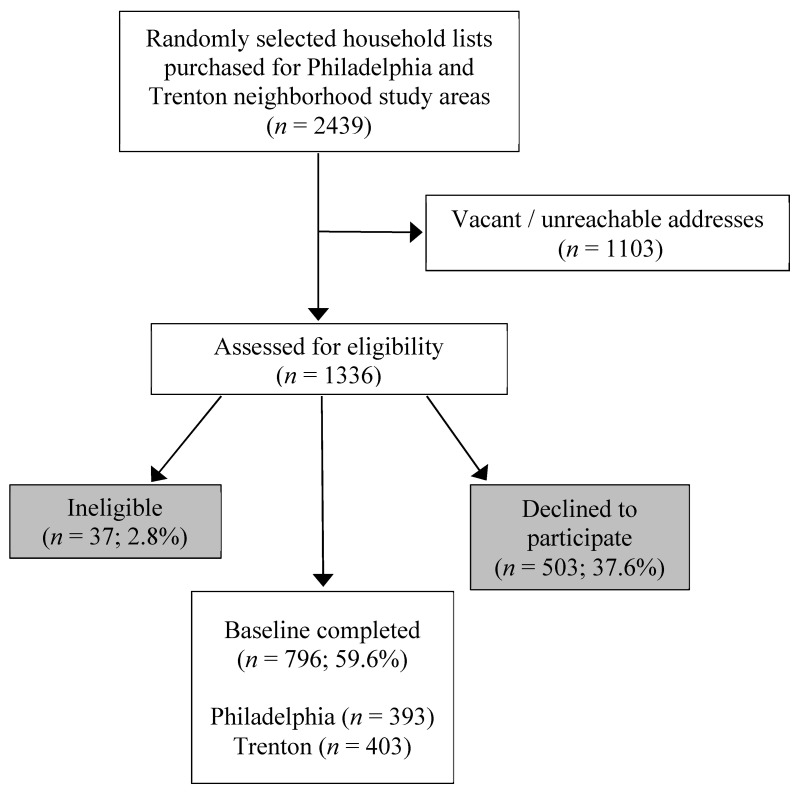
Selection Process for the Food in Our Neighborhood (FIONS) Study Sample.

From the random sample of addresses, participants were recruited using door-to-door (84%) and telephone (16%) methods by trained interviewers. To be eligible for the study, participants had to be 18 years of age or older, speak English or Spanish, be the primary food shopper for the household, and live within one of the study areas. After vacancies, non-responses, ineligibles, and refusals, we enrolled a sample of 796 primary household food shoppers from January through December 2017 (Figure 1).

Ethics approval was provided by the University of Delaware Institutional Review Board, study protocol # 641226 (PI Karpyn). Informed consent was obtained by study staff for all participants.

#### 2.1.1. Participant Surveys (Shopping Preferences, Grocery Spending, Home Food Availability, Perceived Nutrition Environment, and Dietary Outcomes Data)

Study participants responded to an interviewer-administered 65-question survey comprised of ten domains and a total of 120 items. Table 1 outlines survey domains, sources, alignment with previous tests of conceptual models [8,12], and example survey questions. Survey questions were principally sourced from previously validated surveys [8,13,14]. In a few cases, as noted, the research team developed questions based on previously validated surveys. For in-home interviews (84%), heights and weights were measured using procedures from the National Health and Nutrition Examination Survey (NHANES) [15]. For interviews conducted by phone (16%), self-reported heights and weights were recorded.

The tenth survey domain was a 24-h dietary recall using the Automated Self-Administered 24-Hour (ASA24^®^) Dietary Assessment Tool (National Institutes of Health, National Cancer Institute, Bethesda, MD, USA) [16]. As indicated by ASA24^®^ protocol and to reduce bias in dietary assessment, a second 24-h dietary recall was interviewer-administered by phone two weeks after the first data collection. The recalls were conducted on weekdays and weekends. An SAS code from the National Institutes of Health was used to calculate dietary outcome measures from ASA24^®^ dietary assessment data [17]. Dietary outcomes data analyzed were healthy eating index (HEI) score, fruit consumption subscore, and vegetable consumption subscore from participant ASA24^®^ dietary assessments.

The majority of survey items in analyses were ordinal level variables asked on either a four-point or five-point scale. Examples of four-point Likert items were “not at all important” to “very important”, while others were scaled “never/rarely” to “almost always”. All five-point scaled items were on a scale of “strongly disagree” to “strongly agree”. Continuous variables (e.g., age, distance from home to main store, grocery spending, and fruit and vegetable spending) were converted to ordinal variables for easier interpretation and to maintain consistency in item type across factor analysis. Cut points for categories were determined in ways that optimized even distribution of responses across categories.

#### 2.1.2. Store Audits (Observed Nutrition Environment Data)

This study also collected data on observed nutrition environments in supermarkets and corner stores from both study neighborhoods using Nutrition Environment Measures Survey (NEMS) tools. NEMS assessments were conducted in a total of 29 supermarkets and 31 corner stores using NEMS-S and NEMS-CS tools, respectively [18,19]. Both types of retail outlets were scored on availability, price, and quality of both healthier and less-healthy food items.

Audits were conducted at all eligible supermarkets in a two-mile buffer around both study areas as well as in a random sample of corner stores within both three-square-mile study areas. A master store list was compiled in 2016 from Nielsen trade data and publicly available lists of SNAP retailers [20]. Supermarkets were eligible if they were conventional, chain-operated supermarkets, not supercenters or warehouses, had at least two checkout areas, and were within the two-mile buffer around the study areas. Twenty NEMS-S assessments were completed in Philadelphia, out of 21 eligible supermarkets (95%; 1 refusal), while nine NEMS-S assessments were completed in Trenton, out of 11 eligible supermarkets (82%; 1 refusal, 1 missing data).

Corner stores were eligible if they were chain or independent convenience stores, or superettes, located within the study areas. Pharmacies (e.g., RiteAid, CVS) and dollar stores (e.g., Dollar General) were excluded. Among eligible corner stores, a random sample of 22 locations was selected per study area. NEMS-CS assessments were completed in 18 of the 22 corner stores in Philadelphia out of 87 eligible (21%; 4 refusals), while NEMS-CS assessments were completed at 13 of the 22 corner stores in Trenton out of 38 eligible (34%; 9 refusals).

In order to analyze observed nutrition environment scores for individual respondents, and not only for each study area, geospatial and Bayesian statistical methods were used. This resulted in calculation of an estimated NEMS score for each study participant address, based on measured NEMS scores. For chain stores where a NEMS score was calculated for at least one location, we assigned the same or average score to other stores in the same chain. Other stores were assigned the average observed NEMS score for that class of store—i.e., supermarket, Special Supplemental Nutrition Program for Women, Infants, and Children (WIC)-authorized corner store, or corner store not authorized by WIC. We then interpolated a raster surface using the Empirical Bayesian Kriging option in ArcGIS 10.6 Geostatistical Analyst [21] to estimate a NEMS value for each individual participant address. Kriging is a geostatistics method often applied in environmental and earth sciences to predict unknown values based on spatial patterns in sampled data [22]. Estimated NEMS scores were assigned to each participant address using the Extract Values to Points tool in ArcGIS 10.6 Spatial Analyst [21]. NEMS scores ranged from 8.3 to 23.9 (mean = 15.6; SD = 3.4).

#### 2.1.3. Statistical Analyses

Analyses were conducted in two stages: exploratory factor analysis (EFA) and use of a Multiple Indicator Multiple Causes (MIMIC) model.

##### Exploratory Factor Analysis

In the first stage, an EFA was conducted to identify a viable factor structure among over 120 items from participant surveys. We employed EFA over confirmatory factor analysis (CFA) to allow items to load freely onto factors. Utilization of EFA allowed data to define factors based solely on empirical correlations between items. Mplus version 8.3 with default Geomin rotation was used to allow for correlated factor structures [23]. Exploratory factor analyses were repeated until the following criteria were met: (1) items had factor loadings greater than or equal to 0.40; and, (2) items had secondary factor loadings greater than or equal to 0.30. Items that did not meet these criteria were removed one item at a time.

EFA yielded four factors comprised of 22 indicator items. Table 2 shows the four factors, their factor loadings, and corresponding item names that were retained based on goodness of fit statistics. We interpreted factors by examining item content and patterns of coefficients. Items loading onto *My Store’s Quality* (Factor 1) include store cleanliness, availability of fresh foods, and store healthy programs. Items loading onto *Perceptions of Neighborhood Food Availability* (Factor 2) include quality, selection and ease of buying healthy foods in the community. Items loading onto *Neighborhood Safety* (Factor 3) characterize neighborhood satisfaction, walkability and violence. Items loading onto *Household Food Challenges* (Factor 4) reflect availability of unhealthy items in the home, lower grocery and fresh fruit and vegetable expenditures, and transportation barriers.

##### Multiple Indicator Multiple Causes Model

In the second stage of analyses, we extended EFA findings to explore relationships between latent factors and covariates using a Multiple Indicator Multiple Causes (MIMIC) model. The MIMIC model allows for simultaneous evaluation of correlations between multiple latent factors and covariates [24]. Moreover, the MIMIC model allowed us to estimate effects of latent factors on dietary outcome measures (i.e., HEI score, fruit consumption subscore, and vegetable consumption subscore). The MIMIC model process followed required steps: (1) confirming fit of the model using CFA on the 22 items that emerged from EFA; (2) adding covariates to the model to examine their effects on latent factors; and, (3) developing regression models between each latent factor and dietary outcomes (HEI score, fruit consumption subscore, vegetable consumption subscore), while controlling for significant covariates [24].

In final analyses, regression models were developed to examine the extent to which the four latent factors were related to dietary outcomes (HEI score, fruit consumption subscore, and vegetable consumption subscore), while controlling for 11 covariates that remained independently and significantly correlated with latent factors: age, gender, Black/African-American race, Hispanic ethnicity, general health status, physical activity, smoking status, alcoholic drinks per month, SNAP or WIC participation, household income category, and household size (education level and household food insecurity were dropped from the model).

## 3. Results

The study sample reflects demographics of urban, minority and low-income food desert communities. Table 3 shows characteristics of participants by study area. Across the FIONS sample, 60% were African American; 17% were Hispanic; 55% of households had annual incomes less than USD 30,000; 45% reported receiving SNAP or WIC in the past year; 58% experienced at least some food insecurity; 54% had a high school education or less; and mean body mass index (BMI) was in the obese range (mean 30.98). Study participants from Philadelphia and Trenton lived an average of 2.03 and 2.46 miles (median of 1.43 and 1.70 miles, *p* = 0.173), respectively, from the main store where they reported doing major food shopping. Over half the sample (57%) reported getting to their main store by driving their own vehicle.

The four-factor MIMIC model with 22 indicator items and without background characteristics as covariates yielded the following fit indices: *ΔCFI* = 0.99; *ΔTLI* = 0.98; *RMSEA* = 0.05, an excellent fit. Each indicator had a significant relationship at *p* ≤ 0.05 with its corresponding factor. This model emerged from over 120 survey items tested.

The fit of this model plus 11 covariates was similar to the initial MIMIC model (*ΔCFI* = 0.98; *ΔTLI* = 0.98; *RMSEA* = 0.04). The majority of covariates were not statistically significant in the final model, with the following exceptions. Females were more likely to report higher scores on the My Store Quality factor (Factor 1). Identifying as Black/African American and number of people to feed in the household were significantly and directly related to the Household Food Challenges factor (Factor 4). Household income and having a female food shopper for the household were indirectly related with this this factor (i.e., higher income and female shopper households faced less household food challenges).

In examining relationships between four latent factors and three main dietary outcomes, My Store’s Quality and Perceptions of Neighborhood Food Availability (Factors 1 and 2, respectively) had significant positive relationships with vegetable consumption subscore (*β* = 0.11, *p*-value = 0.04; *β* = 0.12, *p*-value = 0.02, respectively). Household Food Challenges (Factor 4) had a significant negative relationship with both HEI score and vegetable consumption subscore (*β* = −0.23, *p*-value < 0.01; *β* = −0.19, *p*-value < 0.01, respectively).

Table 4 shows correlations between the four factors, controlling for covariates. Neighborhood Safety (Factor 3) had statistically significant small to moderate positive correlations with both My Store’s Quality (Factor 1) and Perceptions of Neighborhood Food Availability (Factor 2). These same two factors, My Store’s Quality and Perceptions of Neighborhood Food Availability, were positively correlated to each other. Other correlations were not statistically significant.

**Figure 2:**
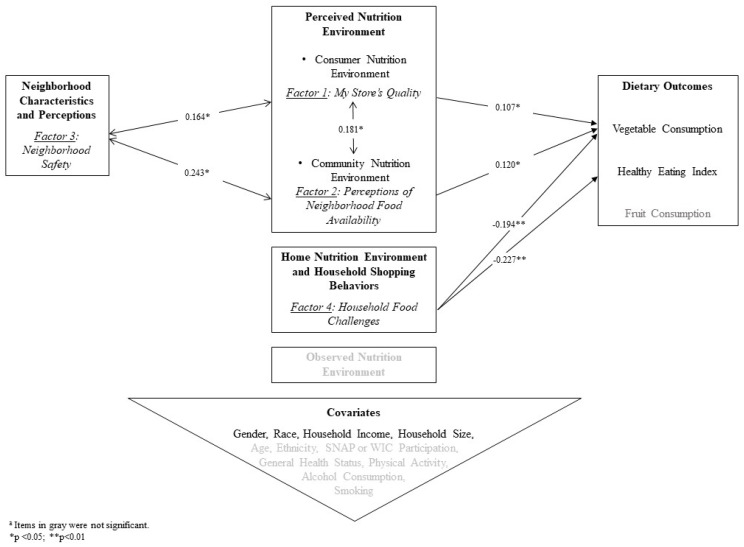
Identified Pathways Among Key Factors Which Contributed to Dietary Outcomes in Urban Food Desert Communities ^a^.

Figure 2 depicts statistically significant factors and confirmatory pathways from this research, as well as alignment with constructs from prior research on the NEMS-P framework [8,12]. This research demonstrates how previous conceptual models of nutrition environments are applicable in urban food desert settings.

## 4. Discussion

This study makes a unique contribution to emerging research on the inter-relationship between perceived and observed nutrition environments, and eating behaviors, as first outlined in frameworks developed by Green and Glanz (2015) and Alber et al. (2018) [8,12]. Alber et al. (2018) found that self-reported perceptions of the food environment were significantly associated with observed measures of the food environment, and that higher perceived prices of fresh fruits and vegetables was moderately associated with BMI [12].

Two latent factors with confirmatory pathways that emerged from this study—perceptions of neighborhood food availability and perceptions of store quality—align with the constructs of perceived community nutrition environment and perceived consumer nutrition environment in the NEMS-P framework. Household Food Challenges (Factor 4) was significantly and negatively associated with both HEI score and vegetable consumption subscore, suggesting more challenging conditions in the home (i.e., less spending per person on groceries, and fruits and vegetables in particular, combined with availability of unhealthy food choices) make it more difficult to achieve a healthy diet. Further, the confirmatory pathway of the Household Food Challenges factor aligns with parallel constructs from the NEMS-P framework, namely home nutrition environment.

Though the Neighborhood Safety factor (Factor 3) did not have a direct statistically significant relationship to dietary outcome scores, it had significant correlations with two other factors that were significantly related to the vegetable consumption subscore. Therefore, Neighborhood Safety is included as a modifier in the summary model of identified pathways (Figure 2).

We found strong confirmatory pathways for perceived nutrition environment factors and dietary outcomes, except for the fruit consumption subscore. Previous research has documented major deficits in fruit consumption among low-income households [25]. Gregory et al. (2019) found that food insecure households consume only about half the fruit that food-secure households do [25]. The overall low levels of fruit consumption and low variability in this outcome among low-income, urban, and minority food desert communities make it difficult to pinpoint risk factors, apart from household income, that are actionable.

NEMS scores estimated for individual participant addresses did not confirm conceptual pathways of the observed food environment, perhaps because of the variability in shopping patterns. Cannuscio et al. (2013) found that, whenever possible, shoppers in underserved areas of Philadelphia chose supermarkets with higher NEMS scores that offered more variety and more healthful options, even when those stores were further away [26]. However, residents of food deserts disproportionately relied on smaller nearby stores with limited food items and unhealthy immediate food environments [26]. Interventions to improve dietary outcomes must address food store proximity as well as making a diversity of healthful foods available. Future analyses can further explore the link between access to a vehicle and shopping at stores with higher NEMS scores.

This research supports refinement of the NEMS-P model given that Neighborhood Safety (Factor 3) emerged as a significant correlate of My Store Quality and Perceptions of Neighborhood Food Availability (Factors 1 and 2, respectively). Previous conceptual work in the urban health field has hypothesized why neighborhood social environment should be considered a critical pathway for obesity prevention [27]. While Neighborhood Safety (Factor 3) was not a significant predictor of dietary quality scores in the current study, its statistically significant correlations with other confirmed factors in the model (Figure 2) suggest that more research is needed to further elucidate these inter-relationships, especially in urban areas.

This study found that the Household Food Challenges factor (Factor 4) was significantly and negatively associated with HEI score and vegetable consumption subscore. The four components of this factor are: (1) lower household grocery spending per person; (2) lower household fruit and vegetable spending per person; (3) more unhealthy food items available in the home in the past week; and, (4) lack of access to a vehicle to get to the household’s main store. Accordingly, this research provides empirical evidence to support previous research that conceptualized these items as predictors of dietary quality. The importance of both grocery and fruit and vegetable spending on dietary quality provides further rationale for healthy food incentive work that is emerging in urban food desert communities to lower prices for fresh fruits and vegetables among low-income households [28,29].

Previous research has shown improvements in perceptions of the healthfulness of a neighborhood food environment following a new supermarket coming into a former food desert community [30]. Perceptions were associated with improved diet, independent of the frequency with which residents shopped at the new store [30]. Future research can further examine these associations given that one of the FIONS study communities received a new full-service supermarket after the first round of data collection [10].

## 5. Limitations

Some limitations of our study should be noted. First, our participant pool reflects urban residents living in two distinct communities within the northeastern section of the United States and may not be representative of other important food desert communities in other areas of the U.S., including rural communities. Further, because we only include residents living in food deserts, findings may appear to minimize the potential importance of the nutrition environment, which in the larger context of healthy food access at a national or regional level, may indeed play a more meaningful role. As such, future analyses should investigate the ways in which the factors tested in this model shift in different contexts. Last, while our survey asked participants about meals eaten away from home, restaurants were not included in our measures of the perceived or observed community nutrition environment.

## 6. Conclusions

This research contributes to the literature on urban, low-income, and minority food desert communities and demonstrates how previous conceptual models of nutrition environments are applicable in these settings. Efforts to improve dietary intake and close gaps in health disparities have focused on communities with limited access to affordable nutritious food, yet few studies have sought to empirically understand the relationship of factors known to impact diet to key dietary outcome measures, specifically among residents experiencing these conditions. Resident perceptions of their nutrition environments and household food challenges (i.e., home availability of unhealthy food and beverages, lower expenditures on fruits and vegetables and groceries, and lack of access to a vehicle) were found to be the most significant factors contributing to dietary outcomes, suggesting a focus for future intervention efforts.

This study confirms the relationship between grocery and fruit and vegetable spending and dietary quality. In the context of low-income communities, this provides a rationale for healthy food incentives and other emerging food policy approaches to increase household income. Poverty and income remain critical determinants of the nutrition environment and ongoing research to understand a broader spectrum of food desert contexts is needed in order to improve generalizability of this model and ultimately inform interventions to close gaps in health disparities.

## Figures and Tables

**Table 1:** Participant Survey Domains, Sources, and Alignment with Conceptual Models of the Nutrition Environment.

Survey Domain	Number of Questions (Items)	Sources	Conceptual Model Alignment [8,12]	Example Questions/Details
1. Main store preferences and perceptions	10 (29 items)	Green and Glanz [8]; Dubowitz et al. [13]	Shopping Behaviors; Perceived Consumer Nutrition Environment	What is the name and address of the main store where you most often do your major food shopping?; How do you usually get to this store?; At [main food store], how hard or easy is it to get [list of food/beverage items]?
2. Grocery spending and household food security	12	8 spending patterns questions developed based on Dubowitz et al. [13]; 2 food insecurity items from Hager et al. [14]	N/A	How much do you spend per month on groceries? How many people does this amount feed? I worried whether our food would run out before we got money to buy more (often true; sometimes true; never true)
3. Perceptions of neighborhood food availability	1 (6 items)	Green and Glanz [8]	Perceived Community Nutrition Environment	It is easy to buy fruits and vegetables in my neighborhood (strongly disagree to strongly agree)
4. Home food availability	4 (31 items)	Green and Glanz [8]	Home Food Environment	Indicate whether each of these food items were available in your home in the past week
5. Neighborhood satisfaction and safety	4	Dubowitz et al. [13]	Neighborhood	I am satisfied with my neighborhood as a place to live (strongly disagree to strongly agree)
6. Demographics	17	Dubowitz et al. [13]	Background Characteristics	Household income and size; race/ethnicity; gender; employment status; vehicle access
7. Participation in food assistance (SNAP and WIC)	6	Dubowitz et al. [13]; 2 WIC questions developed	Background Characteristics	Did any member of your household receive [SNAP / WIC] benefits in the last year?
8. Health status	9 (15 items)	Green and Glanz [8]; Dubowitz et al. [13]	Background Characteristics	Have you ever been told by a doctor that you have any of the following conditions? [list]; Tobacco/alcohol use; level of physical activity
9. Height and weight	2	NHANES procedures [15]	Weight	Self-reported if surveys conducted by phone
10. 24-h dietary recall	ASA24^®^ tool	ASA24^®^ Dietary Assessment Tool [16]	Eating Behaviors	ASA24^®^ (administered twice, 2 weeks apart) for dietary outcomes data (HEI score, fruit subscore, and vegetable subscore [17]

**Table 2:** Factors, Items, and Factor Loadings Identified in Exploratory Factor Analysis of FIONS Participant Survey ^a^.

Factor and Item Descriptions	Factor Loadings
FACTOR 1: My Store’s Quality (8 items)	
Store Cleanliness Score	0.727
Store Availability of Fresh Meats Score	0.713
Store Availability of Fresh Fruits and Vegetables Score	0.682
Store Staff Friendliness Score	0.605
Store Prices Score	0.557
Store Signs to Encourage Healthy Foods Score	0.552
Store Programs to Help Me Buy Healthy Foods Score	0.443
Store Difficulty Getting Lean Meats Score	−0.433
FACTOR 2: Perceptions of Neighborhood Food Availability (6 items)	
Low-fat Products in My Neighborhood are High Quality	0.901
Large Selection of Fruits and Vegetables in My Neighborhood	0.895
Large Selection of Low-fat Products in My Neighborhood	0.892
Easy to Buy Fruits and Vegetables in My Neighborhood	0.871
Easy to Buy Low-fat Products in My Neighborhood	0.867
Fruits and Vegetables in My Neighborhood are High Quality	0.865
FACTOR 3: Neighborhood Safety (4 items)	
I Feel Safe Walking in My Neighborhood During the Evening	0.810
I Am Satisfied with My Neighborhood as a Place to Live	0.703
Violence is a Problem in My Neighborhood	−0.659
I Often Walk Places in My Neighborhood	0.466
FACTOR 4: Household Food Challenges (4 items)	
Amount Spent per Month on Fruits and Vegetables (per person; categorical)	−0.712
Amount Spent per Month on Groceries (per person; categorical)	−0.634
Home Availability of Unhealthy Food and Beverage Items Score	0.422
Does Not Drive Own Vehicle to Main Food Store	0.408

^a^ Selection criteria: primary factor loadings ≥ ± 0.40.

**Table 3:** Background Characteristics of Study Participants by Food Desert Area.

Background Characteristics	Total (*n* = 796) ^a^	Philadelphia (*n* = 393)	Trenton (*n* = 403)	
	% or Mean (SD)	% or Mean (SD)	% or Mean (SD)	*p*-Value
**Age (mean)**	46.93 (15.24)	45.38 (14.64)	48.48 (15.69)	0.005
**Gender**				
**Female**	68.93	78.12	59.95	<0.001
**Male**	31.07	21.88	40.05	
**Race/Ethnicity**				
**Black/African American**	60.43	54.96	65.76	0.002
**Hispanic or Latino**	17.09	18.83	15.38	n.s.
**White**	25.00	28.24	21.84	0.037
**Education**				
**<High school**	14.75	12.76	16.71	n.s.
**High school graduate or GED**	38.97	37.76	40.15	
**More than high school**	46.28	49.49	43.14	
**Annual Household Income Category**				
**<USD 10,000**	23.04	18.73	27.65	n.s.
**USD 10,000–30,000**	32.29	31.68	32.94	
**USD 30,000–60,000**	27.45	30.85	23.82	
**>USD 60,000**	17.21	18.73	15.59	
**Household Size**				
**Number of people to feed (mean)**	3.24 (1.94)	3.58 (2.05)	2.91 (1.78)	<0.001
**SNAP or WIC Participation**				
**Participated in either in past year**	45.35	55.06	35.88	<0.001
**Household Food Insecurity**				
**One or both food insecurity** **conditions sometimes or often true**	58.04	54.71	61.29	n.s.
**General Health Status**				
**Poor**	6.28	6.11	6.45	n.s.
**Fair**	31.16	32.06	30.27	
**Good**	38.32	36.90	39.70	
**Very Good**	15.70	16.79	14.64	
**Excellent**	8.54	8.14	8.93	
**Physical Activity Level**				
**Mostly sedentary**	12.81	11.45	14.14	n.s.
**Moderately active**	51.51	53.69	49.38	
**Moderately to very active**	17.59	19.85	15.38	
**Very active (at least 5 days/week)**	18.09	15.01	21.09	
**Current Cigarette Smoker**	36.93	31.81	41.94	n.s.
**Alcoholic Drinks Per Month (mean)**	9.70 (33.24)	9.91 (37.74)	9.49 (28.19)	n.s.
**BMI (mean)**	30.98 (8.11)	31.30 (8.23)	30.66 (7.99)	n.s.

**^a^**Table 3 reflects valid percentages. Data were missing for <2% of the sample for all characteristics listed, except annual household income which was missing (“refused” or “don’t know”) for 12% of the sample.

**Table 4:** Correlation Coefficients for Factors Identified from Exploratory Factor Analysis of FIONS Survey Variables.

Title	My Store’s Quality	Perceptions of Neighborhood Food Availability	Neighborhood Safety	Household Food Challenges
My Store’s Quality	--	--	--	--
Perceptions of Neighborhood Food Availability	0.181 *	--	--	--
Neighborhood Safety	0.164 *	0.243 *	--	--
Household Food Challenges	−0.071	0.079	0.028	--

* *p* < 0.05, two-tailed.
